# Sperm DNA fragmentation and oxidation are independent of malondialdheyde

**DOI:** 10.1186/1477-7827-9-47

**Published:** 2011-04-14

**Authors:** Nassira Zribi, Nozha Feki Chakroun, Henda Elleuch, Fatma Ben Abdallah, Afifa Sellami Ben Hamida, Jalel Gargouri, Faiza Fakhfakh, Leila Ammar Keskes

**Affiliations:** 1 Laboratory of Human Molecular Genetics, Sfax Faculty of Medicine, Avenue Magida Boulila 3028 Sfax, Tunisia; 2 Laboratory of Histology-Embryology, Sfax Faculty of Medicine, Avenue Magida Boulila 3028 Sfax, Tunisia; 3 Regional Center of Blood Transfusion of Sfax, El Ain Road, 3029 Sfax, Tunisia

## Abstract

**Background:**

There is clinical evidence to show that sperm DNA damage could be a marker of sperm quality and extensive data exist on the relationship between DNA damage and male fertility status. Detecting such damage in sperm could provide new elements besides semen parameters in diagnosing male infertility. We aimed to assess sperm DNA fragmentation and oxidation and to study the association between these two markers, routine semen parameters and malondialdehyde formation.

**Methods:**

Semen samples from 55 men attending the Histology-Embryology Laboratory of Sfax Faculty of Medicine, Tunisia, for semen investigations were analysed for sperm DNA fragmentation and oxidation using flow cytometry. The Sperm was also assessed spectrophotometrically for malondialdehyde formation.

**Results:**

Within the studied group, 21 patients were nonasthenozoospermic (sperm motility ≥ 50%) and 34 patients were considered asthenozoospermic (sperm motility < 50%). A positive correlation was found between sperm DNA fragmentation and oxidation (p = 0.01; r = 0.33). We also found a negative correlation between sperm DNA fragmentation and some sperm parameters: total motility (p = 0.001; r = -0.43), rapid progressive motility (type a motility) (p = 0.04; r = -0.27), slow progressive motility (type b motility) (p = 0.03; r = -0.28), and vitality (p < 0.001; r = -0.65). Sperm DNA fragmentation was positively correlated with coiled tail (p = 0.01; r = 0.34). The two parameters that were found to be correlated with oxidative DNA damage were leucocytes concentrations (p = 0.01; r = 0.38) and broken neck (p = 0.02; r = 0.29). Sperm MDA levels were negatively correlated with sperm concentration (p < 0.001; r = -0.57), total motility (p = 0.01; r = -0.35) and type a motility (p = 0.03; r = -0.32); but not correlated with DNA fragmentation and DNA oxidation.

**Conclusions:**

Our results support the evidence that oxidative stress plays a key role in inducing DNA damage; but nuclear alterations and malondialdehyde don't seem to be synchronous.

## Background

Infertility affects around 15% of couples in reproductive age and male factor is a major contributor by approximately half of these cases [1]. Along with the conventional semen parameters, new tests have been developed to better investigate the pathophysiology and aetiology of male infertility. The role of oxidative stress as a major cause of male infertility has been well established. In fact, reactive oxygen species (ROS) attack all cellular compounds including membrane polyunsaturated fatty acids, proteins, and nucleic acids [2,3]. Detection of such damage in sperm could provide new elements besides semen parameters in diagnosing male infertility. Oxidative stress is assessed using a variety of methods based on the measurement of relatively stable peroxidation products which include three major groups: lipid peroxidation products, oxidised proteins, fragmented DNA or DNA oxidation biomarkers [4].

Lipid peroxidation is one of the deleterious effects of ROS and is considered as an indicator of membrane polyunsaturated fatty acid oxidation [5-8]. Malondialdehyde (MDA) assay is a simple tool used in monitoring such damage. Its outcome correlates well with other techniques for assessing peroxidation including chemiluminescence and colorimetric reactions [8], despite having some drawbacks. These latter are minor when placed against the high sensitivity and convenience of the method [8]. Also this assay has proved to be useful in male infertility diagnosis [8-10].

Moreover, it was reported that ROS, in addition to reacting with the polyunsaturated fatty acids, might also react with DNA nucleotides leading to base modifications particularly 8-hydroxy-2'-deoxyguanosine (8-oxoguanine) formation and DNA fragmentation [2,11-13]. The first type of damage is often referred to as oxidative DNA damage [14,15], and evidence suggests that it is mediated most likely by nitric oxide, superoxide ions, and hydroxyl radicals [15]. The oxidative DNA biomarker 8-oxoguanine is commonly used to evaluate oxidative DNA alterations due to its high specificity and sensitivity, relative abundance in DNA, and potent mutagenicity [14,16]. Detection of this oxidized DNA base remains the best direct assessment of sperm DNA oxidative damage [8]. The second type of damage (DNA fragmentation) was largely studied regarding its relationship with sperm quality and its impact on reproductive outcomes; however results remain controversial [11-13] and the exact molecular mechanisms underlying DNA fragmentation in human spermatozoa remain poorly understood. Numerous tests have been introduced to analyse sperm DNA fragmentation among which Terminal deoxynucleotidyl transferase (Tdt) mediated dUTP Nick End Labelling (TUNEL), COMET (or SCGE, single cell gel electrophoresis), SCSA (Sperm Chromatin Structure Assay), and SCD (Sperm chromatin dispersion) are the most used [11,13].

In this study we aimed to evaluate the concentration of sperm MDA and the levels of DNA fragmentation and oxidation in infertile men and to investigate the eventual correlations between routine semen parameters, sperm DNA damage and malondialdehyde formation.

## Methods

### Patients

This study was approved by the Institutional Review Board of Sfax Faculty of Medicine, Tunisia. A total of 55 men attending the Histology-Embryology Laboratory of Sfax Faculty of Medicine (Tunisia) for semen investigations were included in this study. Written informed consent was obtained from all the subjects for publication of this case report. The patients neither had any urogenital diseases or infections nor did they undergo x- ray or chemotherapy. They were aged between 26 and 62 years old with a mean age (± SD) of 37.49 ± 0.89 years. According to the World Health Organization (WHO) criteria [17], semen samples were classified as nonasthenozoospermic (sperm motility ≥ 50%) or asthenozoospermic (sperm motility < 50%).

### Semen analyses

Semen samples were obtained by masturbation into sterile containers after 3-5 days of sexual abstinence and left to liquefy at 37°C. Basic semen analyses were performed within1 hour of collection and consisted in the measurement of semen volume, sperm concentration (hemocytometer method), motility (total motility, rapid progressive (type a), slow progressive (type b), non progressive (type c)), vitality and morphology. Semen samples were also assessed for leucocytes concentration using peroxidase method. All parameters were carried out according to the WHO guidelines [17].

Sperm DNA fragmentation and oxidation were evaluated in fresh semen. The remainder of the sample was aliquoted and stored at -80°C for further analysis of malondialdheyde levels.

### TUNEL Assay

For the evaluation of DNA fragmentation, a commercial kit (In situ Cell Death Detection Kit, Fluorescein, Roche, Germany) based on an enzymatic reaction of labelling free 3'-OH termini was used. As previously described [16], 3.10^6 ^cells were washed with phosphate-buffered saline (1xPBS, pH 7.4) then fixed with 200 μl of 4% paraformaldehyde for 1 hour at room temperature in the dark. Afterwards, sperm cells were washed with 1 × PBS and permeabilized using 0.1% Triton X-100 in 0.1% sodium citrate for 15 minutes on ice. After washing with PBS, sperm DNA was labelled by incubating spermatozoa with 50 μl of the TUNEL reaction mixture (Tdt enzyme and FITC-labelled nucleotides) in a humidified atmosphere for 60 minutes at 37°C in the dark, with mixing each 15 minutes. Washed and labelled sperm cells were then resuspended in 1xPBS for flow cytometry analysis. A negative control (sample without the addition of Tdt enzyme) and a positive control (sample treated with DNase I (3U/ml, Invitrogen) for 10 minutes at room temperature to generate DNA strand breaks) were also assessed by TUNEL assay.

### Assessment of oxidative DNA damage by Flow Cytometry

We used the oxyDNA kit (Biotrin International, Ireland) which is specific for detection of 8-oxoguanine as one of the major studied oxidised nucleotides. The test is based on the direct binding of a probe conjugated to fluorescein isothiocyanate (FITC-conjugate) to DNA adduct 8-oxoguanine. In brief, as previously described [16,18], one aliquot of each semen sample containing 3.10^6 ^spermatozoa was washed with 1xPBS, fixed, permeabilized with ice-cold 70% ethanol and kept 1 hour at -20°C. Fixed cells were centrifuged at 1600 rpm for 5 minutes, washed with PBS, then resuspended in 1 ml wash solution (Tris-buffered saline/Tween 20 containing thrimerosal) and pelleted at 1600rpm/min for 5 minutes. Fifty μl FITC conjugate were incubated for 1 hour with pelleted sperm cells in the dark at room temperature, with mixing every 15 minutes. Finally, cells were washed, resuspended in 500 μl 1xPBS for flow cytometry analysis. For the positive control, sperm cells were washed in 1ml PBS then incubated in H2O2 (4 M) solution for 1 hour at 37°C. The negative control consisted of selected spermatozoa from healthy men. This control had a percentage of labelled sperm < 2%.

### Flow Cytometry and data analyses

**Figure 1:**
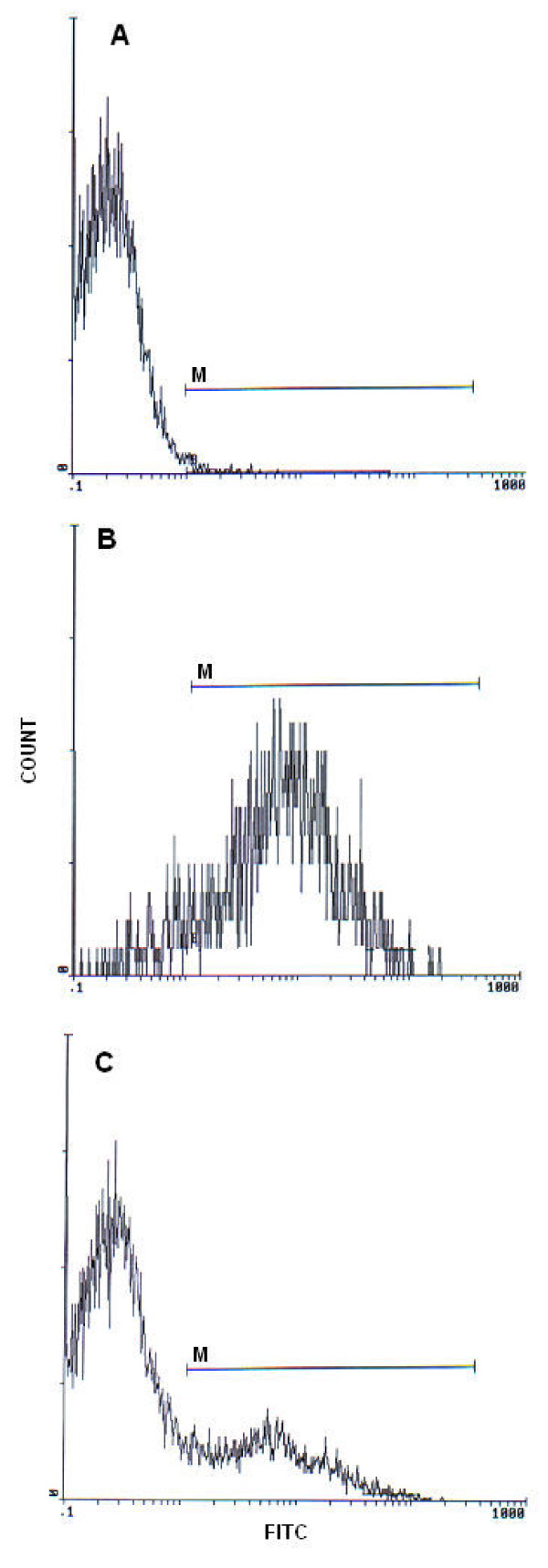
**TdT (terminal deoxynucleotidyltransferase)-mediated dUTP nick-end labeling (TUNEL) assay of spermatozoa**. Histograms show: (A) negative control with 1.35% TUNEL positive cells. (B) Positive control (spermatozoa treated with DNaseI) with 90.2% TUNEL positive cells. (C) Semen sample of one patient with 21.4% TUNEL positive cells. M: window adjusted to detect the percentage of TUNEL positive cells.

**Figure 2:**
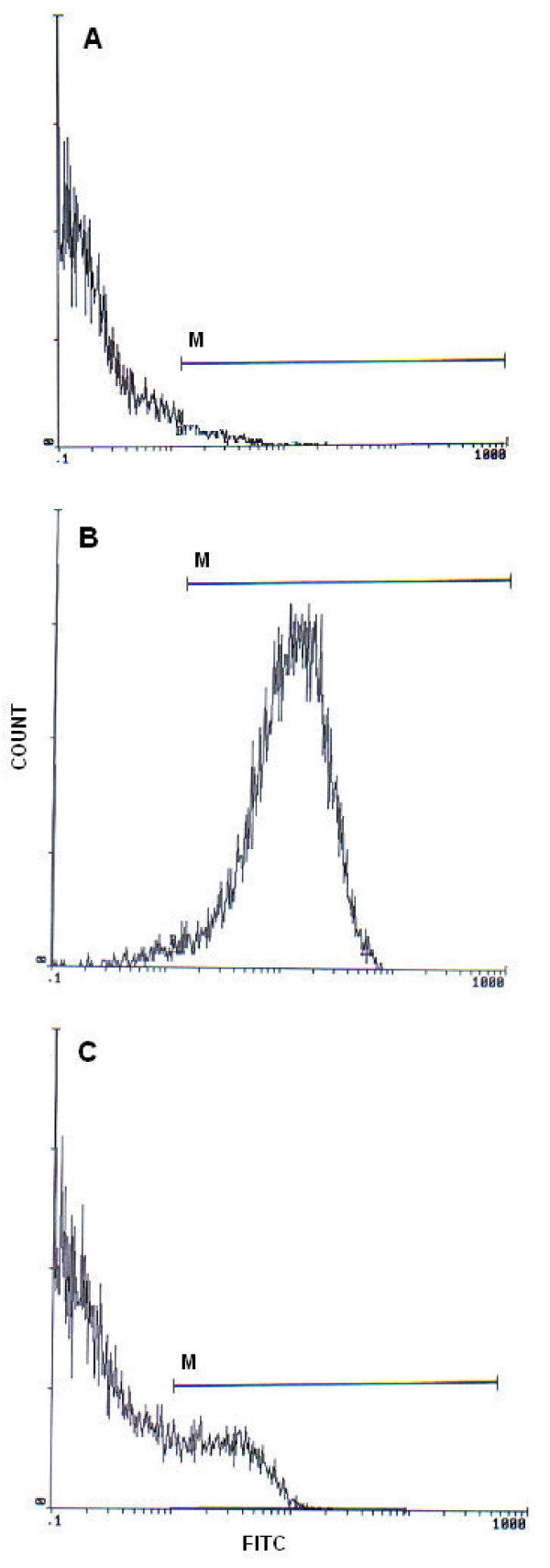
**Flow cytometric 8-oxoguanine detection histograms**. (A) Negative control with 1.95% FITC labelled cells. (B) Positive control with 96.3% FITC labelled cells. (C) Semen sample of one patient with 13.7% FITC labelled cells. M: window adjusted to detect the percentage of DNA oxidation in sperm cells.

Flow cytometric analysis was carried out using an EPICS XL flow cytometer (Beckman Coulter) equipped with a 15mW argon-ion laser for excitation at 488 nm. At least 10000 events per sample were analysed. Light-scattering and fluorescence data were obtained at a flxed gain setting in logarithmic mode. Debris was excluded by establishing a region around the population of interest on the basis of light scatter characteristics (forward-angle light scatter (FSC) vs. side-angle light scatter (SSC). The percentage of labelled sperm was characterized by identifying a region that included > 90% of events in the frequency histogram of the positive controls both in the assessments of DNA fragmentation and oxidation (Figures 1 and 2 respectively). Data were expressed as percentage of stained cells from histograms using System II software. Typical examples of histograms obtained by flow cytometry with markers (M) for the detection of fluorescence are shown in Figure 1 (TUNEL assay) and Figure 2 (8-oxoguanine detection).

### Measurement of lipid peroxidation

Lipid peroxidation in spermatozoa was measured in 43 semen samples with the commonly used thiobarbituric acid reactive substances (TBARS) method, according to Yagi [19]. It was not measured in the remaining 12 semen because of reduced volume or sperm count. Sperm cells in samples prepared for MDA measurement was adjusted to 10^7^. Briefly one part of each sample was added to two parts of TBA reagent (15% v/v trichloroacetic acid and 0.25 N HCl). The mixture was then treated in a boiling water bath for 15 min. After cooling, samples were centrifuged at 4,000 × g for 10 min. The content of MDA was measured spectrophotometrically by the determination of the supernatant absorbance at 532 nm. The MDA fluorescence intensity of spermatozoa was determined using various concentrations of tetraethoxypropane as standards. The results were expressed as nmol MDA/10^7 ^cells. The MDA assay used had an intra-assay coefficient of variation of 7.4%.

### Statistical analysis

The SPSS (SPSS Inc., Chicago, IL) software (version 18) was used for statistical analyses. Non-parametric test (Mann-Whitney U-test) was performed to compare sperm parameters and flow cytometry data between nonasthenozoospermic and asthenozoospermic semen. The relationship between semen parameters, DNA fragmentation, DNA oxidation and MDA levels was analysed using Spearman's correlation coefficients. We applied linear regression to determine the correlation between leucocytes concentration and sperm damage parameters (DNA fragmentation and oxidation and MDA) with adjustment of sperm motility and concentration. Statistical significance was established for a p-value of < 0.05.

## Results

The mean values (± SD) and ranges of routine semen parameters, MDA levels and percentage of sperm DNA damages are summarized in Table 1. Within the studied group, 21 semen were considered nonasthenozoospermic and 34 were considered asthenozoospermic semen. TUNEL-coupled flow cytometry results are expressed as percentage of DNA fragmented sperm cells. Figure 1 presents frequency distribution histograms of negative control (Figure 1A), positive control (Figure 1B), and of a test semen sample (Figure 1C). Figure 2 illustrates the results of DNA oxidation. Histograms show the percentages of sperm with DNA oxidation in negative control (Figure 2A), positive control (Figure 2B), and in a sperm sample from one patient (Figure 2C).

**Table 1:** Summary statistics of semen parameters in the study population (n = 55)

	Mean ± SD	Range
Sperm concentration (.10^6^/mL)	83.7 ± 10.92	2.1 - 456
Sperm motility (%)	42 ± 1.54	0 - 60
Type **a **motility ^a ^(%)	13.64 ± 1.07	0 - 30
Type **b **motility ^a ^(%)	23.09 ± 0.92	0 - 35
Type **c **motility ^a ^(%)	5.45 ± 0.23	0 - 10
Vitality (%)	73.36 ± 1.5	38 - 92
Morphology (%)	8.13 ± 1.07	0 - 41
Leucocytes concentration (.10^6^/mL)	0.43 ± 0.18	0.06 - 9.9
Sperm MDA concentration (nmol/10^7 ^sperm)	7.21 ± 0.72	0.87 - 18.95
Sperm DNA fragmentation (%)	24.64 ± 1.77	3.2 - 67.7
Sperm DNA oxidation (%)	14.35 ± 1.16	1.95 - 48.8

^a ^Grade of sperm movement according to WHO criteria: **a**: rapid progressive motility **b**: slow progressive motility, **c**: non progressive; MDA: Malondialdehyde.

The means (± SD) of sperm MDA concentrations and of DNA fragmentation percentage were significantly higher in the asthenozoospermic group than in nonasthenozoospermic one (8.81 ± 1.01 vs 4.99 ± 0.76 8.81 ± 1.01 nmol/10^7 ^sperm; p = 0.008 and 28.01 ± 2.23% vs 19.34 ± 2.55%; p = 0.006 respectively). However, the levels of 8-oxoguanine (± SD) were not significantly different when comparing the two groups (14.63 ± 1.5% vs 13.92 ± 1.88%; p = 0.8) respectively.

Numerous significant correlations were found between basic semen parameters, sperm MDA levels and DNA damages (Table 2).

**Table 2:** Correlations between semen parameters, MDA levels, DNA fragmentation and DNA oxidation (n = 55)

	MDA	Sperm Concentration	Total motility	Type a motility	Type b motility	Vitality	Leucocytes concentration	Normal morphology	DNA fragmentation	DNA oxidation
MDA	-	**p < 0.001** **r = -0.57**	**p = 0.01** **r = -0.35**	**p = 0.03** **r = -0.32**	p = 0.9 r = 0.01	p = 0.9 r = 0.01	p = 0.9 r = 0.09	p = 0.82 r = -0.03	p = 0.5 r = -0.1	p = 0.7 r = -0.06
Sperm Concentration		-	**p = 0.01** **r = 0.34**	**p < 0.001** **r = 0.53**	p = 0.1 r = -0.2	p = 0.9 r = -0.01	p = 0.53 r = 0.08	p = 0.08 r = 0.23	p = 0.05 r = 0.26	p = 0.1 r = 0.17
Total motility			-	**p < 0.001** **r = 0.82**	**p = 0.001** **r = 0.44**	**p < 0.001** **r = 0.58**	p = 0.9 r = 0.01	**p = 0.002** **r = 0.40**	**p = 0.001** **r = -0.43**	p = 0.8 r = 0.02
Type a motility				-	p = 0.98 r = -0.00	**p = 0.002** **r = 0.4**	p = 0.45 r = 0.10	**p = 0.001** **r = 0.42**	**p = 0.04** **r = -0.27**	p = 0.8 r = 0.01
Type b motility					-	**p = 0.004** **r = 0.38**	p = 0.21 r = 0.12	p = 0.19 r = 0.17	**p = 0.03** **r = -0.28**	p = 0.9 r = -0.01
Vitality						-	p = 0.83 r = 0.28	p = 0.43 r = 0.10	**p < 0.001** **r = -0.65**	p = 0.6 r = -0.06
Leucocytes concentration							-	p = 0.6 r = -0.07	p = 0.32 r = -0.13	**p = 0.01** **r = 0.38**
Normal morphology								-	p = 0.69 r = -0.05	p = 0.29 r = -0.14
DNA fragmentation									-	**p = 0.01** **r = 0.33**
DNA oxidation										-

Significant correlations (p and r values): bold characters; MDA: Malondialdehyde.

**Figure 3:**
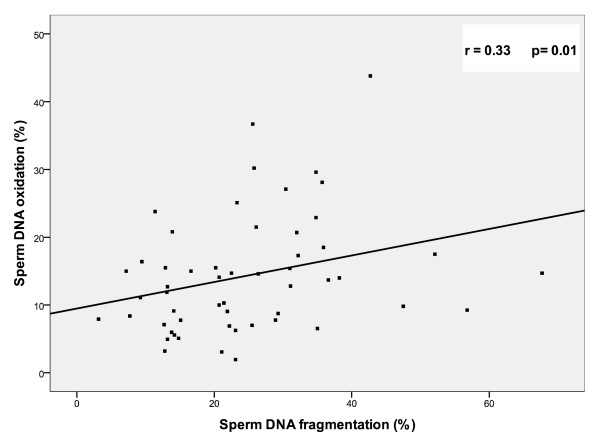
**Correlation between sperm DNA fragmentation and sperm DNA oxidation (n = 55)**.

In fact, sperm DNA fragmentation was positively correlated with sperm DNA oxidation (p = 0.01; r = 0.33) (Figure 3). Concerning correlations between DNA fragmentation and routine semen parameters: TUNEL assay correlated negatively with sperm total motility (p = 0.001; r = -0.43); the same negative but weaker correlations were found with type a motility (p = 0.04; r = -0.27) and type b motility (p = 0.03; r = -0.28); however, a strong correlation was noted with sperm vitality (p < 0.001; r = -0.65). Sperm DNA fragmentation was positively correlated with coiled tail (p = 0.01; r = 0.34). The two parameters that were found to be correlated with oxidative DNA damage were leucocytes concentrations (p = 0.01; r = 0.38) and broken neck (p = 0.02; r = 0.29). Sperm MDA levels were negatively correlated with sperm concentration (p < 0.001; r = -0.57), total motility (p = 0.01; r = -0.35) and type a motility (p = 0.03; r = -0.32). MDA was neither correlated to DNA fragmentation (p = 0.5) nor to DNA oxidation (p = 0.7) (Table 2).

## Discussion

Numerous studies have focused on the relationship between sperm DNA damage and standard semen parameters [20-24] but scarce others have searched for clues to its origins [25-28]. Our study is one of the first reports in which flow cytometric TUNEL and OxyDNA assays in combination with the MDA test were employed to investigate the role of oxidative stress in generating sperm DNA damages.

As regards the relationship between sperm DNA damages and semen quality, we found that DNA fragmentation was higher in asthenozoospermic patients than that in nonasthenzoospermic ones. The high prevalence of DNA fragmentation in dyspermic semen samples as compared to normospermic was reported previously [20-24,29]. Sperm DNA fragmentation correlated also with some semen parameters according to other studies [28-32]. Regarding DNA oxidation, we detected no significant difference between the two study groups however; 8-oxoguanine levels were correlated with leucocytes concentration and with one of sperm morophological abnormalities (broken neck). These findings are in concordance with some reports [33] but in disagreement with others [18,34-36]; this controversy could be due to the use of different methods that lack standardization and validation in different subject populations [37]. For instance, the detection of 8-oxoguanine was carried out using HPLC [35,36], ELISA [33] and in recent reports flow cytometry [18,26,28].

Besides the evaluation of sperm DNA fragmentation and oxidation, we used the malondialdehyde (MDA) assay to measure lipid peroxidation in spermatozoa. This assay was chosen, first for its simplicity and sensitivity [38]; Secondly because the association between poor sperm quality and high MDA concentration was shown in many previous studies [33,38,39]; whereas, only few reports [33,40] were interested in studying the relationship between MDA levels and sperm DNA damage, particularly 8-oxoguanine [33]. In the study of Nakamura et al [33], authors did not find a significant correlation between 8-oxoguanine, as measured by ELISA, and malondialdehyde concentrations in seminal plasma. In another report [40], MDA content was shown to be linked to sperm DNA decondensation but not with DNA fragmentation. Also, based on a meta-analysis of published results [4], Dotan et al, reported that malondialdehyde was correlated to several peroxidation products including F2-Isoprostanes, lipid hydroperoxides, conjugated dienes, glutathione and protein carbonyls; but not with DNA fragmentation products (using comet assay) and antioxidants concentration.

As expected, our results showed that sperm MDA concentration was significantly higher in asthenozoospermic than in nonasthenzoospermic patients. Besides, we found that MDA levels were negatively correlated to sperm concentration and motility which was consistent with previous reports [39,40]. In other studies, it was reported that peroxidation reaction affects membrane structure and fluidity and causes damage to axonemal proteins leading to a permanent impairment in sperm motility [2,3,38].

Among the interesting findings of our study, a significant correlation was observed between sperm DNA fragmentation and oxidative DNA damage. This result was similar to that reported in a recent study by Aitken et al., [28] suggesting a link between the two types of DNA damage and supporting the recently published data arguing in favour of a ROS attack on sperm DNA [26-28,41]. Nevertheless, we did not find a correlation between sperm DNA alterations (oxidative DNA damage and sperm DNA fragmentation) and lipid peroxidation as assessed by MDA assay. Although these two types of sperm damage are oxidative in origin, it was suggested that there are two independent steps in generating sperm DNA damage and lipid peroxidation [40]. The lack of correlation between sperm DNA damage and MDA levels reported in the present study could be also due to some technical considerations. In fact, as proposed by Mitchell et al [42], it would have been interesting to use a reducing agent like DTT to relax sperm chromatin and give the detection reagents (used in OxyDNA and TUNEL assays) access to the internal DNA structure regarding the highly compact nature of sperm chromatin.

The second important result we reported for the first time was the significant correlation between leucocytes concentration and 8-oxoguanine. However, leucocytes concentration was correlated neither to DNA fragmentation nor to MDA levels. This makes us suggest a direct implication of leucocytes, as an exogenous factor, in generating base modifications, and a more susceptibility of DNA bases to ROS action; obviously, 8-oxoguanine formation reflects a direct and specific action of ROS on sperm DNA [8]. It seems also that the plasma membrane is less vulnerable to oxidative damage than DNA since at certain levels of ROS, sperm with significant oxidative DNA damage could retain the ability to fertilize probably because their membranes are still intact [43]. Our data showed also correlations between DNA damages and some morphological abnormalities. It was suggested that sperm cells themselves especially morphologically abnormal spermatozoa may generate ROS which could induce sperm DNA damage [44,45].

From literature data, many hypotheses were postulated to explain the origin of sperm DNA damage such as defective chromatin packaging during spermiogenesis [11,43,46], aberrant or abortive apoptosis before ejaculation and oxidative stress [8,11,12]. The latter plays a major role in generating sperm DNA fragmentation; firstly because high levels of ROS were correlated to DNA single and double strand breaks [2,43]; secondly, sperm DNA damage induced by the hydroxyl radical or after exposure to ionizing radiation was associated with the formation of 8-oxoguanine in a first stage and followed by single-stranded DNA breaks [12,14]. Recently, it was suggested that aberrant spermatogenesis could lead to alterations in chromatin packaging and a deficiency in protamination which would make sperm DNA more susceptible and vulnerable to a variety of stressors, mostly the ROS action [27]. In addition, it was hypothesised that activation of caspases and endonucleases which is triggered by cytochrome C release from mitochondria and mediated largely by ROS could induce sperm DNA fragmentation [12]. Nevertheless, Aitken et al [27] postulated that the physical architecture of spermatozoa could prevent these nucleases from translocating to the nucleus and suggested that DNA fragmentation could result from nonenzymatic reaction or from the action of activated endonuclease already integrated into the chromatin body. The most recent studies on the origin of sperm DNA damage suggested that there might be a cascade of changes that progress from the induction of oxidative stress and oxidized DNA base adduct formation to DNA fragmentation and cell death [28].

Sperm damages could be also caused by different events related to iatrogenic, idiopathic, environmental and pathological factors and to lifestyle [8]; however, it was not possible to study the impact of such factors since the patients included in our study neither had urogenital diseases, infections or medication treatments nor did they undergo x- ray or chemotherapy.

Sperm DNA damage is thought to be detrimental to ART outcomes, particularly to pregnancy rates [12,13,31,28]. Moreover, it was shown that oxidative sperm DNA damage influences fecundity [47] and that a higher percentage of spermatozoa with 8-oxoguanine was associated with a lower embryo quality after IVF or intracytoplasmic sperm injection [18].

Finally, it should be noted that the techniques used to assess sperm DNA integrity need more validation and standardization in order to better understand the nature and the causes of DNA abnormalities in human spermatozoa [37,48]. In recent studies the proposed modified protocols, particularly for TUNEL assay seemed to be simpler and more robust [42,49].

## Conclusions

The present study suggests a link between DNA fragmentation and oxidative base damage but lipid peroxidation seems to be an independent sperm decay although all of these alterations are linked to oxidative stress. Free radicals-induced sperm damage has been studied extensively and there is evidence showing that a significant proportion of the DNA damage observed in human spermatozoa is oxidative in nature. However, other molecular mechanisms underlying such damage need to be elucidated by further studies that should take into consideration the major role of oxidative stress in causing sperm DNA damage. In addition, the use of antioxidants could have beneficial effects in preventing such damage and ameliorating semen parameters and reproductive outcomes [1].

## Competing interests

The authors declare that they have no competing interests.

## Authors' contributions

NZ made the work of conception, methodology, design, and acquisition of data and statistics, analysed and interpreted the data, drafted and revised the manuscript, tables and figures, and revised the manuscript. NFC contributed to the conception and design, analysed and interpreted the data, and revised the manuscript. HEE and JG carried out the flow cytometry analysis and participated in data interpreting. FBA participated in lipid peroxidation measurement and in statistical analyses. FF and LAK revised the manuscript critically for important intellectual content and final approval. All authors read and approved the final manuscript.
